# Early detection of communication delays with the PEDS tools in at-risk South African infants

**DOI:** 10.4102/ajod.v5i1.223

**Published:** 2016-04-08

**Authors:** Jeannie van der Linde, De Wet Swanepoel, Linique Hanekom, Tasha Lemmer, Karla Schoeman, Frances Page Glascoe, Bart Vinck

**Affiliations:** 1 Department of Speech-Language Pathology and Audiology, University of Pretoria, South Africa; 2 Ear Sciences Centre, School of Surgery, The University of Western Australia, Nedlands, Australia Ear Science Institute Australia, Subiaco, Australia; 3 School of Medicine, Vanderbilt University, United States of America; 4 Department of Speech-Language Pathology and Audiology, Ghent University, Belgium

## Abstract

**Background:**

Prevalence of communication delays or disorders is increasing, possibly because of various environmental risk factors. Selection and implementation of effective screening tools are important to detect at-risk infants as early as possible. This study aimed to evaluate the accuracy of the Parents’ Evaluation of Developmental Status (PEDS), PEDS-Developmental Milestones and PEDS tools to detect communication delays in infants (6–12 months) in a South African primary healthcare context.

**Method:**

A comparative study design evaluated the accuracy of the PEDS tools to detect communication delays, using an internationally accepted diagnostic assessment tool, the Rossetti Infant-Toddler Language Scale (RITLS). A convenience sample of 201 infants was selected at primary healthcare clinics.

**Results:**

Expressive and receptive language sensitivity scores were low across all three screens (ranging between 14% and 44%). The PEDS tools had high sensitivity (71%) and specificity (73%) ratings for the receptive and expressive language and socio-emotional domain in combination.

**Conclusion:**

In the sample population, the PEDS tools did not accurately detect receptive and expressive language delays; however, communication delays in general were identified. Future research determining accuracy of the PEDS, PEDS-Developmental Milestones and PEDS tools for children aged 2–5 years in detecting communication delays should be prioritised.

## Introduction

The prevalence of communication delays or disorders is increasing and may be ascribed to environmental factors such as unemployment, limited medical resources, lack of educational services, violence, crime and HIV or AIDS (Guralnick 2013). Paediatric HIV or AIDS, for instance, is a challenging condition as it not only influences the well-being of infants but also results in prematurity and low birth weight, and later attention difficulties and speech and language delays (Rossetti 2001; Samuels, Slemming & Balton 2012). South African infants and children are particularly vulnerable because of the high prevalence of predisposing environmental factors such as these (Mayosi & Benatar 2014; Samuels *et al*. 2012).

The high prevalence of developmental delays or disorders amongst infants in South Africa (Samuels *et al*. 2012) necessitates selection and implementation of effective screening or developmental surveillance tools to identify at-risk infants as early as possible (Van der Linde *et al*. 2015). If communication delays remain undetected until primary school years, a child is at greater risk for behavioural problems, academic failure and socio-emotional disturbances (Squires *et al*. 2009; Yew & O’Kearney 2013). With a direct link between school performance, communication skills and the role that communication plays in general development and emotional and behavioural outcomes, the importance of early identification of communication delays is obvious (Rossetti 2001; Wankoff 2011; Yew & O’Kearney 2013). Early identification of and early intervention for infants at risk can prevent or reduce future developmental difficulties and academic failure whilst improving the quality of life for the infant and family (Samuels *et al*. 2012). Furthermore, these services can prevent or lessen developmental and communication difficulties (Hawa & Spanoudis 2014), which implies less future financial expenditure for parents with regard to medical costs, transport fees to medical centres and/or speech therapy expenses.

Infants receiving early intervention services, including early detection by means of developmental screening and/or surveillance as first point of access, make greater progress when the whole family is involved (Guralnick 2013). Because parents are usually the first to identify their children’s developmental difficulties, they are considered a good resource by healthcare providers when conducting screening tests (Williams & Holmes 2004). The resource-constrained public healthcare system in developing countries like South Africa (Mayosi & Benatar 2014) requires time-efficient and accurate screening tools to ensure it is practically feasible with low false-positive rates that do not result in over-referral. Parents can be used as a resource in identifying their child’s strengths and weaknesses (Glascoe 2013), and providing important information to professionals. A parent-administered test may therefore be appropriate for the South African context if it is sufficiently accurate and time efficient. Furthermore, selecting a comprehensive screening tool that accurately detects communication delays in addition to other developmental delays may be more suitable than developmental domain–specific screening tools in the South African, resource-constrained public healthcare context.

Early identification of developmental delays, including communication delays, can be facilitated by a variety of valid standardised tools. In South Africa, developmental screening is implemented nationally as part of the Road to Health Booklet (Tarwa & Villiers 2007). However, the Road to Health Booklet has not been validated and its accuracy for developmental screening has been questioned (van der Linde *et al*. 2015). The Ages and Stages Questionnaire or ASQ (Squires *et al*. 2009), Denver Developmental Screening Test II (Frankenburg *et al*. 1992) and the Parents’ Evaluation of Developmental Status or PEDS (Glascoe 1997) are all well validated and standardised screening tools with large bodies of supporting evidence (Macy 2012). All three tools include infants from birth; however, the Denver Developmental Screening Test II is a clinician-administered test, whereas the ASQ and PEDS tools are parent-administered tools (Macy 2012).

The ASQ and the PEDS elicit parental concerns regarding their children’s development and behaviour. In a comparison study conducted in Canada, both the ASQ and PEDS rendered similar outcomes and it was concluded that either one can be selected for implementation (Limbos & Joyce 2011). Taking into consideration the cost of the tools and administration time, the PEDS tools have been deemed more appropriate for use in the South African primary healthcare (PHC) context.

Whilst a recent study evaluated the accuracy of the PEDS and PEDS-Developmental Milestones (PEDS-DM) for developmental delays in the private healthcare sector in South Africa (Silva 2010), the accuracy of the PEDS test detecting communication delays or disorders in infants in the South African PHC context has not yet been established. This study therefore evaluated the accuracy of the PEDS tools in detecting communication delays in infants, aged 6–12 months, in a PHC context in South Africa.

## Method

A comparative cross-sectional within-subject design was employed to evaluate the accuracy of the PEDS tools in detecting communication delays using the Rossetti Infant-Toddler Language Scales (RITLS) as a gold standard.

### Setting

Data were collected at three PHC clinics, namely Olievenhoutbosch Clinic, Salvokop Clinic and Daspoort Polyclinic. These clinics are situated in underserved communities in the Tshwane District, Gauteng Province, South Africa. The community in Olievenhoutbosch consists of 70 863 individuals and 23 777 households. The clinic serves an area of 11.39 km² and is situated in Centurion (Statistics South Africa 2011). Daspoort covers an area of 2.16 km², with 6355 individuals and 1582 households (Statistics South Africa 2011). Salvokop has a population of 7123 individuals and 1685 households within an area of 4.09 km² (Statistics South Africa 2011).

### Participants

As this study focused on early identification, infants between 6 and 12 months of age were targeted. Convenience sampling was used as all caregivers of infants between 6 and 12 months proficient to communicate in English or Afrikaans were asked to participate. The sample consisted of 201 infants, and the caregiver of each was interviewed.

### Material

Because the current study aimed at evaluating the accuracy of the PEDS, PEDS-DM and PEDS tools in detecting communication delays, the RITLS (Rossetti 2001) were used as the gold standard reference. It is a comprehensive, easy-to-administer and relevant tool that was designed to assess the preverbal and verbal aspects of interaction and communication in the young child (Rossetti 2001). Although this is a criterion-referenced tool, it has been used and validated in previous studies (Desmarais *et al*. 2010; Dettman *et al*. 2007; Groenewald, Kritzinger & Viviers 2013; Rie, Mupuala & Dow 2008; Steiner *et al*. 2012; Sylvestre & Mérette 2010). The RITLS assesses interaction-attachment, pragmatics, gestures, play and language comprehension and expression of infants from birth to 3 years (Rossetti 2006).

The PEDS tools, that is the PEDS and PEDS-DM, consist of questions posed to the parent/caregiver. The PEDS consists of 10 questions that address parental concerns about their infant’s development. The tool can be conducted either as a questionnaire, in which parents write down their responses, or as an interview, where the healthcare professional asks the questions. It includes the following domains: cognition, expressive and receptive language, gross and fine motor, self-help, academic, health, socio-emotional/mental status and behaviour (Glascoe 2013). Each of these areas is represented irrespective of the child’s age (birth to 7 years 11 months) and is time- and cost-effective (Glascoe 2013). The tool takes approximately 5 minutes for parents to complete and approximately 1–2 minutes for the healthcare professional to score (Glascoe 2013) with a clear score guide and algorithm for referral (Glascoe 1997). The referral algorithm consists of five paths, namely Paths A–E:

- Path A – When two or more predictive concerns about self-help, social, school or receptive language skills are present, refer to the respective allied healthcare professional.
- Path B – When one predictive concern is present, administer the second-stage developmental screen, if second screen is failed refer.
- Path C – When non-predictive concerns are present, counsel in areas of difficulty and follow-up.
- Path D – When parental difficulties are present in communicating because of foreign language barrier, use translator in second screen.
- Path E – When no concerns are present, re-screen at next visit.

Furthermore, in Path B distinction is made between development-related predictive concerns and health-related concerns.

The PEDS-DM consists of six questions posed to parents regarding their infant’s or child’s developmental milestones. The six questions differ in each age interval and represent the following areas of development: fine motor, receptive language, expressive language, gross motor, self-help and socio-emotional.

### Procedures

The PEDS tools and RITLS were administered by an experienced speech–language therapist in a screening environment that was secluded and had limited distractions and low noise levels. The procedure entailed fetching the caregiver and infant from the clinic, obtaining informed consent, completing the assessment and interview and providing feedback. The infants were assessed according to their chronological age. Referral letters for follow-up services were provided when necessary. This process took approximately 30–45 minutes to complete. Appreciation for participating in the study was shown by providing a meal for the infant.

### Data processing and interpretation

#### Rossetti Infant-Toddler Language Scale

Information obtained through elicitation, observation and by report from caregivers carried equal weight when scoring the RITLS (Rossetti 2001). If a specific behaviour was not elicited, observed or reported, it indicated that the infant had not yet reach the expected age level. The subtests are divided into 3-month intervals, for example 0–3 months, 4–6 months and 7–9 months. When the developmental level is two intervals or more below the infant’s chronological age, the infant is considered delayed (Rossetti 2001). For example, if an infant is 10 months of age, but scores on a 0- to 3-month-old level in the Play subsection. It is important to note that the Gesture subsection only starts at the 9- to 12-month interval. Therefore, none of the infants could present with a delay in this developmental area.

### PEDS tools

The PEDS was interpreted in the following manner: Path A–D was deemed a fail and Path E was deemed a pass (Glascoe 2013). If an infant had one or more unmet milestone in the PEDS-DM, the outcome of the test is a fail. The interpretation of the PEDS tools started with the PEDS, where Path A represented a fail irrespective of the PEDS-DM result, but with Path B–E, the PEDS-DM results determined the actual pass or fail.

### Data analysis

The SAS version 9.3 was used to conduct the data analysis. The pass/fail and delayed/not delayed distributions and percentages were calculated. The pass/fail distribution of the PEDS, PEDS-DM and PEDS tools and the delayed/not delayed distribution of the RITLS were presented separately in two-way tables for each domain, that is receptive language, expressive language and socio-emotional. The socio-emotional outcomes of the PEDS, PEDS-DM and PEDS tools were compared against the interaction-attachment subtest of the RITLS. The domain-specific sensitivity, specificity and positive and negative predictive values of the PEDS, PEDS-DM and PEDS tools were then calculated.

## Results

### Participants’ profile

The average age of the 201 infants (45% female infants) was 8.7 months (SD 1.9; range 6–12 months). Fifteen different home languages were reported, of which Sepedi (33%), isiZulu (16%) and Shona (11%) had the largest representation. All participants were proficient in either English or Afrikaans as an additional language, but none reported either of these as their home language. Most of the individuals resided in Olievenhoutbosch (94%). The remaining 6% were from other areas in Tshwane such as Mamelodi and Salvokop. The majority of the participants were black (98.5%). Seven of the 201 infants were from teenage pregnancies, and 6 infants were born prematurely. Of the total sample, 62% of the parents did not complete their high school education, 71% of the households had a monthly income of R3000 or less and 32% had three or more children in the home.

### Fail rates of the PEDS screening tools and RITLS

A positive diagnosis of communication delay was made for 13% (*n* = 26) of the entire sample (see Table 1). Almost half (47%; *n* = 94) of the sample failed the PEDS on one or more of the general developmental domains, and 65% (*n* = 17) of these failed screens were also identified as having a communication delay on the RITLS. Similar fail rates were obtained with the PEDS-DM (49%; *n* = 98) and PEDS tools (52%; *n* = 104). Domain-specific fail rates are also presented in Table 1.

**TABLE 1:** Fail rates of the screening tools and RITLS.

Variable	PEDS (%)	PEDS-DM (%)	PEDS tools (%)	RITLS (%)
Overall	47 (94/201)	49 (98/201)	52 (104/201)	13 (26/201)
Receptive language	3 (6/201)	8 (16/201)	10 (20/201)	4 (9/201)
Expressive language	3 (7/201)	7 (15/201)	10 (21/201)	11 (22/201)
Social-emotional	9 (19/201)	11 (22/201)	19 (38/201)	1 (2/201)
Combined*	12 (25/201)	22 (45/201)	32 (65/201)	12 (24/201)

RITLS, Rossetti Infant-Toddler Language Scale; PEDS, Parents’ Evaluation of Developmental Status; PEDS-DM, PEDS-Developmental Milestones.

* Receptive and expressive language and social-emotional skills.

### Accuracy of the screens in detecting communication delays

Because the PEDS, PEDS-DM and the PEDS tools are developmental screening tools that include various developmental aspects, domain-specific results were compared to the RITLS; focusing only on the accuracy of the tools in detecting communication delays (see Table 2).

**TABLE 2:** Developmental domain-specific performance of the PEDS tools in comparison to the RITLS.

Developmental Domain	PEDS (%)	PEDS-DM (%)	PEDS tools (%)
**Receptive language**			
Sensitivity	22 (2/9)	33 (3/9)	44 (4/9)
Specificity	98 (188/192)	93 (179/192)	92 (176/192)
Positive predictive values	33 (2/6)	19 (3/16)	20 (4/20)
Negative predictive values	96 (188/195)	97 (179/185)	97 (176/181)
**Expressive language**			
Sensitivity	5 (1/22)	23 (5/22)	23 (5/22)
Specificity	97 (173/179)	94 (169/179)	91 (163/179)
Positive predictive values	14 (1/7)	33 (5/15)	24 (5/21)
Negative predictive values	89 (173/194)	91 (169/186)	91 (163/180)
**Social-emotional**			
Sensitivity	100 (2/2)	50 (1/2)	100 (2/2)
Specificity	91 (182/199)	89 (178/199)	82 (163/199)
Positive predictive values	11 (2/19)	5 (1/22)	5 (2/38)
Negative predictive values	100 (182/182)	99 (178/179)	100 (163/163)
Combined*			
Sensitivity	25 (6/24)	58 (14/24)	71 (17/24)
Specificity	90 (158/177)	82 (146/177)	73 (129/177)
Positive predictive values	24 (6/25)	31 (14/45)	26 (17/65)
Negative predictive values	90 (158/176)	94 (146/156)	95 (129/136)

RITLS, Rossetti Infant-Toddler Language Scale; PEDS, Parents’ Evaluation of Developmental Status; PEDS-DM, PEDS-Developmental Milestones.

* Receptive and expressive language and social-emotional skills.

The sensitivity of both the receptive and expressive developmental domains was poor in the PEDS (22% and 5%), PEDS-DM (33% and 23%) and the PEDS tools (44% and 23%). Receptive language sensitivity was higher than expressive language sensitivity in all three tests. The specificity, however, in both domains were high (between 89% and 98%). Similarly, the positive predictive value was poor (between 14% and 33%), in contrast to a high negative predictive value (between 89% and 97%). The PEDS tools’ combined sensitivity, that is receptive and expressive language and socio-emotional domains, was 71% with the combined specificity being 73%.

## Discussion

The fail rates of the PEDS, PEDS-DM and PEDS tools were high (47% – 52%). This was to be expected as an at-risk population was used. Several high-risk factors for developmental delay were present in the study population. The majority of participants had one or more risk factor(s) for developmental delays, such as poverty (71%), three or more children in a home (32%) and limited parental education (62%). An estimated 45% of the South African population is poor, whilst 20% live in extreme poverty (Statistics South Africa 2011). Multiple risk factors increase the probability that development will be delayed (Glascoe & Leew 2010) and high-risk children are 24 times more prone to have IQs below 85 than low-risk children (Sameroff *et al*. 1987).

Specificity and sensitivity values of an accurate screening tool should fall between 70% and 80% (Glascoe 2013). The results in this study demonstrated domain-specific (i.e. expressive language and receptive language) sensitivity scores that were low to very low across the PEDS, PEDS-DM and PEDS tools. Such low sensitivity values may result in a failure to identify a large number of infants who require early communication intervention services. The PEDS tools, on the other hand, did show an accurate sensitivity (71%) and specificity (73%) rating for receptive and expressive language and socio-emotional domains in combination. High sensitivity and specificity for socio-emotional developmental delays indicated that the infant delays in the study sample were accurately identified by means of the PEDS and PEDS tools. Autism spectrum disorders, for example are characterised by such impairments in social interaction, communication and behaviour, which are ostensible before the age of 3 years (Baio 2012). Because the results of this study indicated that PEDS and PEDS tools are able to accurately detect socio–emotional developmental delays in infants, these tools may possibly aid in the early diagnosis of autism spectrum disorders in PHC.

The lack of parental concern regarding their infants’ communication development in the current study population, as illustrated by the fail rate of the PEDS for receptive (3%) and expressive language (3%), were similar to previous research findings. A study performed by Glascoe (2013) revealed that parents of infants, 11 months or younger, do not have many communication-related concerns. However, when there are concerns, it usually pertains to their children’s motor, health, behavioural, self-help and socio-emotional skills (Glascoe 2013). This is possibly because gross motor milestones, such as sitting and crawling, are more observable than infant’s speech sounds and language comprehension (Glascoe 2013).

The low sensitivity and specificity ratings of the screening tools for receptive (22% – 44%) and expressive language (5% – 23%) reported in the current study are likely because of the difficulty to identify communication delays before the age of 12 months (Eadie *et al*. 2010). It can be expected that parents’ awareness of their child’s communication development might be better at a later stage when the child is older and more communicative (Eadie *et al*. 2010). It is therefore recommended that future research should evaluate the accuracy of the PEDS tools for communication delays in 2- to 5-year-old children within the South African PHC context. Because the interviews and assessments were not conducted in the home languages of the sample population, it may be deemed a limitation of the current study. Future research should explore the accuracy of translated tools in detecting communication delays in infants and young children. Preventative strategies, such as developmental surveillance and awareness campaigns, should be considered as a way to support underserved communities where the majority of infants are at risk of communication and/or other developmental delays.

## Conclusion

The PEDS tools demonstrate limited sensitivity scores for receptive and expressive language domains in young infants, although sensitivity for the socio-emotional domain was high. Obtained values for the PEDS tools did demonstrate a high degree of accuracy when considering a combination of receptive and expressive language and socio-emotional domains with sensitivity and specificity of 71% and 73%, respectively. Future research determining accuracy of the PEDS, PEDS-DM and PEDS tools for children aged 2–5 years in detecting communication delays should be explored.
